# How to improve patient education on deep brain stimulation in Parkinson’s disease: the CARE Monitor study

**DOI:** 10.1186/s12883-017-0820-7

**Published:** 2017-02-21

**Authors:** Lars Dinkelbach, Bettina Möller, Karsten Witt, Alfons Schnitzler, Martin Südmeyer

**Affiliations:** 1 Department of Neurology and Institute of Clinical Neuroscience and Medical Psychology, Heinrich Heine University, Düsseldorf, Germany; 2 Department of Neurology, University of Kiel, Kiel, Germany; 3 Department of Neurology, Ernst von Bergmann Klinikum, Charlottenstrasse 72, Potsdam, D-14467 Germany

**Keywords:** Deep brain stimulation, Parkinson’s disease, Treatment acceptance, Patient education, Referral

## Abstract

**Background:**

The introduction of deep brain stimulation (DBS) about 25 years ago provided one of the major breakthroughs in the treatment of Parkinson’s disease (PD). However, a high percentage of patients are reluctant to undergo DBS. Previous research revealed that the critical step on the patient’s path to DBS is the decision whether to undergo further diagnostic assessment for surgery at a specialized DBS-center. The aims of the current study were to evaluate how effective the combination of an outpatient DBS screening tool, STIMULUS, with specially developed educational material was to enhance patient education on DBS and to identify motivational aspects which influenced the patients’ willingness to undergo further assessment.

**Methods:**

In total, 264 patients were identified as appropriate candidates for DBS by general neurologists using the electronic preselection tool STIMULUS. Patient-centered information material was designed and handed out to support education on DBS. Further, several clinical characteristics and details of the patient counseling were documented. Refusal or consent to show up at a DBS center was registered over the following 16 months.

**Results:**

114 (43.2%) patients preselected as eligible for DBS (STIMULUS Score ≥ 6) agreed to show up at a specialized DBS center to undergo further diagnostic assessment. The patients’ ages, PD classification as an akinetic-rigid type and the talks’ topics side-effects of dopaminergic medication and the optimal time frame had a significant influence on the patients’ decisions.

**Conclusions:**

The combination of preselection tools as STIMULUS with comprehensive information material is effective to increase DBS-acceptance rate in PD patients. Important topics of the information about DBS cover the optimal time frame for DBS surgery, the side-effects of dopaminergic medication as well as side-effects and complications of DBS surgery.

**Electronic supplementary material:**

The online version of this article (doi:10.1186/s12883-017-0820-7) contains supplementary material, which is available to authorized users.

## Background

Chronic DBS is a well-tolerated, beneficial and widely established treatment for Parkinson’s disease [1, 2]. The selection process usually consists of two steps: A general neurologist preselects the patients and refers them to a specialized DBS center which takes the final decision for or against surgery based on comprehensive diagnostic assessment.

Bearing in mind that the risk of surgery rises with age while the benefit tends to decrease [3, 4], the right timing for a recommendation to undergo DBS treatment is crucial and merely depends on the evaluation by the general neurologist. Nevertheless, the decision seems to be a struggle for general neurologists as only 48 to 55% of the patients initially referred to DBS centers were later assessed as appropriate candidates for surgery [5, 6]. In order to support the preselection, Moro and colleagues developed an online screening tool called STIMULUS [7]. This tool aids the general neurologists in the referral of patients with a range of registered demographic and clinical parameters. The application of STIMULUS could decisively improve the preselection process as 77% of referred patients were later assessed as appropriate candidates by the DBS center [6].

Despite the high accuracy for preselection, this study raises another issue: After 6 months, only 28% of patients with referral as recommended by STIMULUS actually showed up at the DBS center, mainly due to patients’ reluctance to undergo surgery [6]. During further assessment in the DBS center, only 6% of patients were excluded for reasons such as poor motivation [6].

The need for patient information provided by professionals is highlighted by a questionnaire study, addressing the attitude of Parkinson patients and their relatives towards DBS [8]. The study suggests that the main factors leading to refusal were unrealistic doubts and mixed expectations of this treatment [8], as a result of insufficient information provided predominantly by the media or other patients [8, 9].

## Methods

The aim of the CARE Monitor study was to improve patient education for DBS and to identify key factors that influence the patients’ decision to undergo further diagnostic assessment at a specialized DBS-center. For this purpose, 51 general neurologists located all over Germany were trained on how to operate the STIMULUS screening tool and to use a newly developed information material. Approval from the ethics committee of the Heinrich Heine University, Düsseldorf was obtained (N: 4641). All data were collected anonymously.

### Construction of the information material

Based upon patients’ doubts and expectations as revealed by a questionnaire study [8], a comprehensive information booklet was developed to support physicians in educating the patients about DBS. In 26 pages, the benefits, risks and right timing of DBS as well as its procedure for Parkinson’s disease are described. A DVD was attached to the booklet, including a 4-minute film illustrating the DBS procedure for treating Parkinson’s disease. The German version of the booklet as well as the film are available at http://www.medtronic-caremonitor.de.

### Data collection

**Fig. 1:**
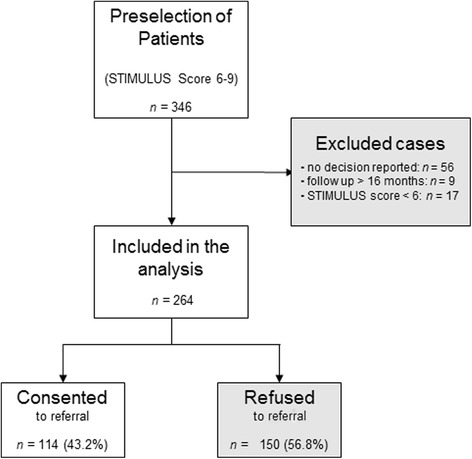
Illustration of the screening and preselection process. 264 patients were preselected as promising candidates for neurostimulation and thus included in the current study. Finally, 114 (43.2%) of 264 preselected patients consented to referral to a specialized DBS-center to undergo further diagnostic examinations

In the following, patients with a STIMULUS score from 6 to 9 were identified as promising candidates for DBS by general neurologists. Figure 1 illustrates the preselection process. In total, general neurologists registered 346 patients from January 2010 to May 2012. From these initial reports, 264 (age 63.7 ± 9.5, 92 female) patients met the criteria as promising candidates for DBS and were therefore included in the study. These patients were recommended for referral to a DBS-center to undergo further diagnostic assessments and were comprehensively informed about this treatment, supported by the newly developed information material. The general neurologists were free to choose the topics dealt with during patient counseling but were asked to document these by filling out a questionnaire. In addition, several patients’ data, such as age, gender, their disease characteristics and the role of the information material were recorded. For an English version of the questionnaire, see the Additional file 1.

### Data analyzation

In view of the exploratory nature of our study, we decided to perform a stepwise approach to identify the predictive factors leading to patients’ consent. First, Mann-Whitney U tests (for ordinal and scaled data), Chi-Square tests (for nominal data with more than two categories) and Fisher’s exact tests (for nominal data with two categories) were applied to identify candidate variables with a potential influence on patients’ decisions. Subsequently, variables with a significant relationship to patients’ decisions (*p* < 0.05) were stated as candidate variables and thus included in a stepwise binary logistic regression. For categorical data, dummy variables were created. Due to this stepwise approach, we could ensure that at least ten outcome cases were provided for each predictor variable [10]. All analyses were conducted using IBM SPSS Statistics 22 (International Business Machines Corporation, Armonk, USA).

## Results

Within 16 months of the initial consultation visit, 114 (43.2%) out of 264 preselected patients (STIMULUS Score ≥ 6) consented to show up at a specialized DBS center. The newly designed information material was well accepted and stated to be “helpful” by 156 (78.4%) patients (“uncertain” *n* = 28, 14.1% “not helpful” *n* = 15, 7.5%, *n* = 65 missing values). Accordingly, the patients’ evaluation of the information material had a highly significant correlation with patients’ decisions (*p* < 0.001).

For a comprehensive overview of all analyzed variables see Table 1. In total, eleven variables were identified as promising predictors of patients’ decisions and therefore underwent binary logistic regression analysis. These were: patients’ age, Parkinson subtype (akinetic‐rigid, tremor‐dominant or equivalent), the total number of topics addressed during the patient counseling and the following contents of the counseling talk: motor improvement, quality of life, side-effects of medication, optimal time frame, evidence of DBS, other topics. Due to the high number of missing values (*n* = 65, 24.6%) patients’ evaluations of the information material were not included in the regression model.

**Table 1:** Correlation between patients’ consent to be referred to a DBS center, clinical characteristics and the contents of clarification sessions

		Group Consent		Group Refusal		
		*N* = 114 (43.2%)		*N* = 150 (56.8%)		
	*N*	*Median*	*25./75. Quartiles*	*Median*	*25./75.Quartiles*	p*
Numerical Data						
Age	262	63	53–70	67	60–71	*0.002*
Disease duration	245	9	5–12	8	6–12	0.618
Hoehn & Yahr Stage	250	3	2–3	3	2–3	0.995
Eligibility for DBS (STIMULUS Score)	260	8	7–9	8	7–9	0.682
Subjective Impairment	255	7	6–8	7	6–8	0.178
Number of topics addressed in the clarification talk	264	6	4–9	5	3–9	*​0.001*
Categorial Data	*N*		*%*	*N*	*%*	p*
Gender	Male	73	64.0	99	66.0	0.795
	Female	41	36.0	51	34.0	
Occupation	Working	21	18.4	17	11.3	0.362
	Retired	79	69.3	118	78.7	
	Unemployed	13	11.4	14	9.3	
Subtype	Akinetic-rigid	45	39.5	33	22.0	*​0.008*
	Tremor dominant	21	18.4	38	25.3	
	Equivalent	48	42.1	79	52.7	
Evaluation of the information material	Helpful	79	89.8	77	69.4	<*​0.001*
	Uncertain	8	9.1	20	18.0	
	Not helpful	1	1.1	14	12.6	
Information source^‡^	Media	4	3.5	9	6.0	0.265
	Doctor	93	81.6	117	78.0	0.539
	Support group	3	2.6	8	5.3	0.360
	Others	1	0.9	1	0.7	>0.999
	Patient with DBS	3	2.6	0	0.0	0.079
Contents of clarification talk^‡^	motor improvement	104	91.2	117	78.0	*0.004*
	quality of life	106	93.0	114	76.0	<*​0.001*
	side effects of medication	86	75.4	76	50.7	<*0.001*
	optimal time frame	66	57.9	50	33.3	<*0.001*
	expectations	60	52.6	62	41.3	0.081
	change of role model in partnership	20	17.5	25	16.7	0.870
	patients fears	20	17.5	20	13.3	0.388
	evidence of DBS	44	38.6	34	22.7	*​0.006*
	experience with DBS	43	37.7	54	30.0	0.191
	complications of DBS surgery	63	55.3	74	49.3	0.385
	side effects of DBS	58	50.9	63	42.0	0.171
	effects of medication withdrawal	32	28.1	39	26.0	0.780
	progression of PD	52	45.6	58	38.7	0.260
	others	16	14.0	9	6.0	*​0.034*

* To analyze the impact of numerical data on patients’ decisions, Mann-Whitney U tests were calculated and the median and 25.-75. quartiles are presented. For categorial variables with more than two categories (subtype and occupation) Chi-Square tests were conducted. For categorial variables with two categories, Fisher's exact tests were conducted. Exact two-tailed p values are presented. A variable with a *p* value < 0.05 was considered as a potential predictive variable and therefore included in the binary regression analysis.

‡ In the categories *information source* and *contents of clarification talk* more than one option could be reported. Therefore, Fisher's exact tests were calculated for the prevalence or absence of each topic/source and the resulting *p* values are presented. To clarify the presentation only the number of prevalent cases are shown.

The entries in italicized represent significant *p* values (lower than 0.05)

The resulting regression model covering 262 cases (two cases were excluded due to missing age documentation) explained 20.5% of the variance (Nagelkerke R^2^, a medium effect according to a common classification [11], *Χ* ^2^ = 43.5, *p* < 0.001) and correctly classified 68.7% of all cases. The following four variables significantly improved the predictive value of the regression model on patients’ decision (*p* < 0.05) and were therefore part of the resulting model: age, classification as an akinetic-rigid type as well as the talk contents dealing with the “side-effects of medication” and “optimal time frame”. None of the other variables could significantly improve the determination of variance and thus were not included in the regression model. For the *p* values and odds ratios of the variables included in the regression model, see Table 2.

**Table 2:** Coefficients of the predictive regression model

	Odds ratio^†^	95% Confidence Interval		*p*
Age	0.96	0.93	0.98	0.002
Akintetic-rigid type	2.32	1.29	4.15	0.005
*Clarification talk contents*				
optimal time frame	2.24	1.28	3.94	0.005
side effects of medication	2.22	1.23	4.0	0.008

Table 2 summarizes the variables which significantly increased the predictive value of the resulting regression model.

† Odds ratios compare patients who consented with patients who failed to show up at a DBS-center. As an example, the Odds ratio of 2.32 indicates that the chance of a “consent” patient having an akinetic-rigid subtype is 2.32 times higher than the chance of a “refuser”

## Discussion

Poor consent to undergo DBS surgery is one of the major problems of this treatment, as only 28% [6] of Parkinson patients consented in the referral to a specialized DBS-center, even after being preselected as promising candidates for this treatment. Thus, a large majority of patients were not reached to undergo a potentially beneficial treatment.

The resulting regression model of our study suggests that the consultation visit to provide information on DBS plays a key role in patients’ decisions, whereas the disease severity as evaluated by the Hoehn & Yahr Stages or subjective impairment as well as the clinical eligibility for DBS measured by the STIMULUS score do not. Of the various topics that could be addressed, the fact that, whether or not the side-effects of dopaminergic medication and the optimal time frame for DBS (meaning rising risks in surgery with age while benefits decrease [3, 4]) were mentioned in the patients’ education had the greatest predictive value for patients’ consent. The relevance of right timing is supported by the significant predictive value of the patient’s age as the rate of consent decreased with age. Interestingly, mentioning negative aspects of DBS during the consultation visit, such as the risks and complications of the surgery or side-effects of the stimulation, had no negative effect on patients’ approval, even in our exploratory initial analysis without a correction for multiple comparisons (see Table 1). Descriptively, a slight trend towards higher approval after mentioning these factors could be noted-a hint, that overdrawn fears of patients are a greater problem than unrealistic expectations, as described in a previous survey [8]. Therefore, we highly recommend that negative aspects of DBS should not be avoided with the intent to not frighten patients. This should rather be an obligatory topic to be addressed during the education for DBS, not least for ethical reasons.

In our study, patients with an akinetic-rigid subtype of Parkinson’s disease had a significantly higher likelihood to consent to the referral to a DBS-center, despite recently published evidence indicating higher benefit of DBS for other subtypes, such as the tremor dominant [12]. Possibly, akinetic-rigid patients are in greater need for alternative treatments as the prevalence of this subtype contributes to a poorer quality of life [13]. On the other hand, we were not able to find a relationship between the patients’ subjective impairment and their decisions. However, we cannot completely rule out a mediating effect of the patients’ quality of life as we did not apply a conventional measurement of quality of life such as the PDQ-39 assessment.

An ongoing discussion addresses the common finding that approximately twice as many men are recipients of DBS than women [14, 15] even though epidemiology studies suggest an equal distribution of Parkinson’s disease [16, 17]. With regard to clinical eligibility for DBS, no gender differences were found, suggesting that non-biological factors may be accountable for this prominent gender discrepancy in DBS recipients [18]. Setiawan and coworkers proposed that women may have more doubts or fears regarding DBS [15]. In line with previous results, the current study could reproduce the typical gender distribution as only 92 (34.8%) women were preselected as eligible candidates for DBS. In contrast to Setiawan and colleagues’ assumption, the patients’ genders were not related to their decision to undergo DBS. Hence, the reasons for the gender distribution of parkinsonian patients undergoing DBS remain unclear.

In our study, 31.8% of the patients consented to the referral within the first 6 months after the initial screening, the same time interval as in Wächter and colleagues’ trial [1] (see Additional file 2:Figure S1). Although this ratio indicates only a slight increase of approval with the usage of information material, it probably underestimates its effect. The current study was designed to follow the patients’ decision for 16 months and the participating neurologists were therefore not obliged to report consent within the first 6 months. In the total 16-month period of the study, 114 (43.2%) of the 264 patients who were preselected as eligible for DBS consented to show up at a specialized DBS-center. Therefore, our data indicates that adequate training of general neurologists and support with information materials for patients and their relatives can increase the rate of approval for DBS treatment. This assumption is supported by the high correlation of patients’ evaluation of the information material to their decisions (see Table 1). Accordingly, our data emphasize the usefulness of information materials such as booklets or DVDs as an effective and low-cost method to support the patient counseling for DBS.

Even though our data clearly indicate a strong relevance of the patient information, no causal connections can be deduced because our study is lacking a control group receiving no or reduced information about DBS. Further, our data only cover a German cohort and thus should be interpreted with caution regarding other health-care systems.

## Conclusions

Adequate patient education by general neurologists is fundamental to allay patients’ aversion to DBS and should cover the optimal time frame to undergo surgery as well as dopaminergic side-effects which could be avoided by DBS treatment. Negative aspects of this therapy should not be evaded and are an obligatory topic in any ethical clarification session. Specialized, patient-centered information material provides a low-cost and effective way to additionally support education about DBS.

## Additional files

Additional file 1: English Version of the Questionnaire. This questionnaire was used to collect all patients’ characteristics and the informative talks’ contents that could be of potential relevance for patients’ decision to undergo further diagnostic assessment in a specialized DBS center. This questionnaire was handed out to 51 general neurologists located all over Germany to track patients’ decisions for 16 months following an initial patient briefing on DBS. (DOC 48 kb)
Additional file 2: Figure S1. Patients’ rate of consent in the course of time. Percentage of patients who consented in the referral after follow-up intervals of up to 16 months. Descriptively, more than a half (*n* = 60) of all consenting patients underwent further diagnostic examinations within the first 3 months after initial referral was suggested. This emphasizes the key role of the informative talk. Afterwards, the gradient of the acceptance rate tends to stabilize. (PNG 18 kb)

## Abbreviations

DBS: Deep brain stimulation
N: Number
PD: Parkinson’s disease
PDQ-39: Parkinson`s Disease Questionnaire
