# Supplementary material for: How to improve patient education on deep brain stimulation in Parkinson’s disease: the CARE Monitor study
Source: BMC Neurol. 2017 Feb 21;17:36. doi: 10.1186/s12883-017-0820-7 (PMC5320695; doi:10.1186/s12883-017-0820-7)
Supplement: Additional file 1: — English Version of the Questionnaire. This questionnaire was used to collect all patients’ characteristics and the informative talks’ contents that could be of potential relevance for patients’ decision to undergo further diagnostic assessment in a specialized DBS center. This questionnaire was handed out to 51 general neurologists located all over Germany to track patients’ decisions for 16 months following an initial patient briefing on DBS. (DOC 48 kb) [file 12883_2017_820_MOESM1_ESM.doc]

CARE Monitor I

Data collection about dedicated patient education for the treatment with DBS (Deep Brain Stimulation)

Consecutive Number: _________________ Date: ______________

Center Address and stamp: ____________________________________________________

ZIP: _______________ State: ____________ Fax Number: _____________________

**Anonymous patient data**

Postal Code (only 3 figures): _ _ _ gender:  m  f Date of birth:

Disease duration of IPS: _______ years employment:  yes  no  retired

**Diagnosis**

Hoehn & Jahr Stage _____  Tremor dominated STIMULUS Score _______

Akinetic rigid type

Equivalent type

**Course of disease**

Number of hospitalizations during the last 12 months: _______

Due to:  fluctuations  medical side effects  other

Grade of subjective impairment 1-10 (10 highest impairment): ________

PDQ 39 (if possible): ________

Medical treatment before DBS therapy:

Levodopa  Dopamine agonist  MOA-B inhibitor  COMT inhibitor  Amantadine

Anticholinergic  Duodenal pump therapy  Apomorphine inusions  Other

Patient received information about DBS via:  Media  support-groups  physician

Other  Patients with DBS

**Consultation visit to inform about DBS**

Patient brochure has been used:  yes  no

Which of the following topics have been addressed?

| motor improvement  quality of life  medication side effects  window of opportunity  expectations (patient and relative)  changing role in partnership  results of patient anxiety survey  evidence (German guidelines) | experience: number of patients worldwide, years of experience  complications of DBS surgery  side effects due to stimulation  side effects due to drug withdrawal  disease progression  Other |
| --- | --- |

**Follow-up visit (latest 16 months after information consultation)**

Date of follow-up visit: ___________ Number of months after consultation: ____________

Did you find the training material helpful?  yes  no  uncertain

What are the key factors (according to priority) that influenced your decision?

______________________________________________________________________________

Patient agrees to DBS therapy:  yes  no

Referred at: ________ day/ months/ year

Name of DBS center: _____________________________
