# Supplementary figures and images for: How to improve patient education on deep brain stimulation in Parkinson’s disease: the CARE Monitor study
Source: BMC Neurol. 2017 Feb 21;17:36. doi: 10.1186/s12883-017-0820-7 (PMC5320695; doi:10.1186/s12883-017-0820-7)

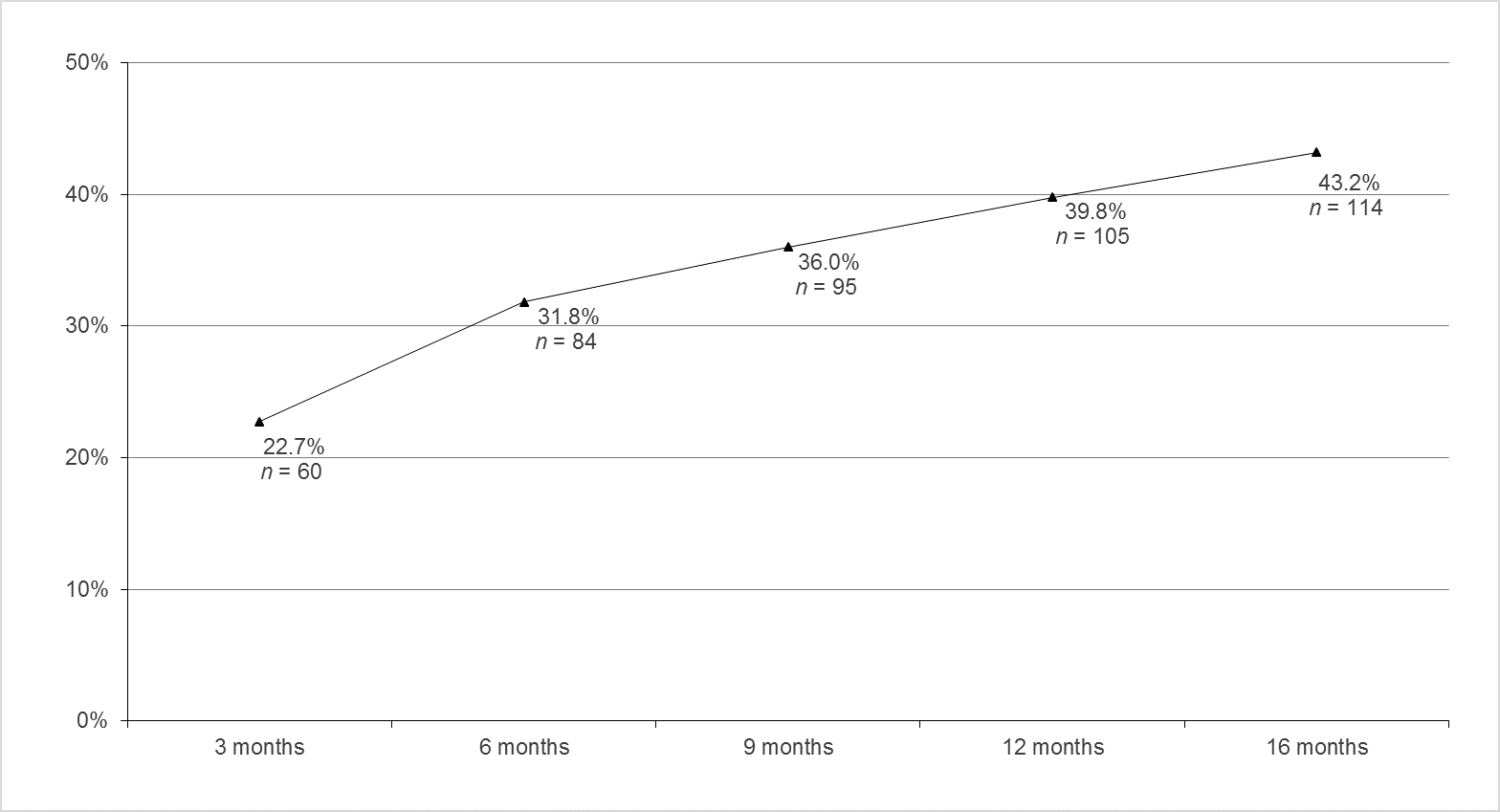

Supplement: Additional file 2: Figure S1. — Patients’ rate of consent in the course of time. Percentage of patients who consented in the referral after follow-up intervals of up to 16 months. Descriptively, more than a half (n = 60) of all consenting patients underwent further diagnostic examinations within the first 3 months after initial referral was suggested. This emphasizes the key role of the informative talk. Afterwards, the gradient of the acceptance rate tends to stabilize. (PNG 18 kb) [file 12883_2017_820_MOESM2_ESM.png]
